# Sexual Dysfunction in Psychiatric Patients: Prevalence and Associated Factors

**DOI:** 10.1192/j.eurpsy.2026.12117

**Published:** 2026-07-31

**Authors:** S. Aouadi, A. Aissa, Y. Ben Othman, G. Ltaeif, R. Hosni, R. Jomli

**Affiliations:** 1 Avicenne Psychiatry; 2 Psychiatry, Hospital Razi, Tunis, Tunisia

## Abstract

**Introduction:**

Sexual dysfunction (SD) is common in psychiatric patients but remains underdiagnosed. Contributing factors include patient reluctance to discuss the issue and a lack of systematic assessment by healthcare professionals.

**Objectives:**

To determine the prevalence of SD in psychiatric patients and identify associated factors.

**Methods:**

A descriptive and analytical cross-sectional study was conducted in the Avicenne Psychiatry Department at Razi Hospital in Tunis between January and March 2025, including stabilized outpatients. Data were collected using a pre-established form and the Psychotropic-Related Sexual Dysfunction Questionnaire (SALSEX).

**Results:**

Our study included 50 patients, predominantly male (75%), with a mean age of 41 years (range: 22–66 years). Forty-two percent were married, and the majority (68%) resided in urban areas. Eighty-seven percent had not completed education beyond the secondary level, while 58% were unemployed and of low socioeconomic status.

With regard to lifestyle habits, 52% of participants were smokers, 10% reported alcohol consumption, and 23% used illicit substances.

In terms of medical and psychiatric history, 87% had no significant organic comorbidities. The most frequent psychiatric diagnoses were bipolar disorder (45%), schizophrenia (29%), and schizoaffective disorder (23%). The majority (84%) were receiving antipsychotic treatment, and 39% were on mood stabilizers or combination therapy.

Overall, 45% of patients presented with sexual dysfunction (SD), of which 23% was moderate and 16% severe in intensity. Among those affected, 57% were in a relationship, and 38% reported marital conflicts; however, no significant association was found between marital conflict and SD (p = 0.569).

Regarding sexual education, 36% had received no formal sexual education, while 64% reported having received education framed within a taboo context. Nonetheless, no significant relationship was observed between the type of sexual education and SD (p = 0.226).

Concerning pharmacotherapy, 72% were treated with atypical antipsychotics, and 50% were receiving a concomitant mood stabilizer. Specifically, 43% were on lithium, 36% on olanzapine, and 21% on risperidone.

**Conclusions:**

This study reveals that nearly half of psychiatric patients experience SD. Although many factors may contribute, no significant correlations were established with the factors studied. Therefore, it is crucial to adopt a systematic screening approach to ensure appropriate treatment and comprehensive management of risk factors.

**Disclosure of Interest:**

None Declared

